# Geospatial data for peer-to-peer communication among autonomous vehicles using optimized machine learning algorithm

**DOI:** 10.1038/s41598-024-71197-6

**Published:** 2024-08-30

**Authors:** T. M. Aruna, Piyush Kumar, E. Naresh, G. N. Divyaraj, K. Asha, Arunadevi Thirumalraj, N. N. Srinidhi, Arunkumar Yadav

**Affiliations:** 1 Department of AIML, Nitte Meenakshi Institute of Technology, Bengaluru, India; 2 Department of AIML and IPR Cell, Nitte Meenakshi Institute of Technology, Bengaluru, India; 3 Department of Information Technology, Manipal Institute of Technology Bengaluru, Manipal Academy of Higher Education, Manipal, India; 4 Department of AIML, Nitte Meenakshi Institute of Technology Bengaluru, Karnataka, India; 5 School of Computer Science and Technology, Reva University, Bengaluru, India; 6 Department of Computer Science Engineering, K. Ramakrishnan College of Technology, Trichy, Tamil Nadu India; 7 Department of Computer Science and Engineering, Manipal Institute of Technology Bengaluru, Manipal Academy of Higher Education, Manipal, India; 8 Department of Civil Engineering, Manipal Institute of Technology Bengaluru, Manipal Academy of Higher Education, Manipal, India

**Keywords:** Artificial intelligence, Support vector machine kernel, Elephant herding optimization, Grey wolf optimizer, Autonomous vehicles communication, Civil engineering, Environmental impact, Environmental sciences, Solid Earth sciences, Engineering

## Abstract

The transportation infrastructure of the future will be based on autonomous vehicles. When it comes to transportation, both emerging and established nations are keen on perfecting systems based on autonomous vehicles. Transportation authorities in the United States report that driver error accounts for over 60% of all accidents each year. Almost everywhere in the world is the same. Since the idea of self-driving cars involves a fusion of hardware and software. Despite the rapid expansion of the software business and the widespread adoption of cutting-edge technologies like AI, ML, Data Science, Big Data, etc. However, the identification of natural disasters and the exchange of data between vehicles present the greatest hurdle to the development of autonomous vehicles. The suggested study primarily focused on data cleansing from the cars, allowing for seamless interaction amongst autonomous vehicles. This study's overarching goal is to look at creating a novel kind of Support Vector Machine kernel specifically for P2P networks. To meet the kernel constraints of Mercer's theorem, a newly proposed W-SVM (Weighted-SVM) kernel was produced by using an appropriately converted weight vector derived through hybrid optimization. Given the advantages of both the Grey Wolf Optimizer (GWO) and the Elephant Herding Optimisation (EHO), combining them for hybridization would be fantastic. Combining the GWO algorithm with the EHO algorithm increases its convergence speed, as well as its exploitation and exploration performances. Therefore, a new hybrid optimization approach is proposed in this study for selecting weights in SVM optimally. When compared to other machine learning methods, the suggested model is shown to be superior in its ability to handle such issues and to produce optimal solutions.

## Introduction

**Fig. 1:**
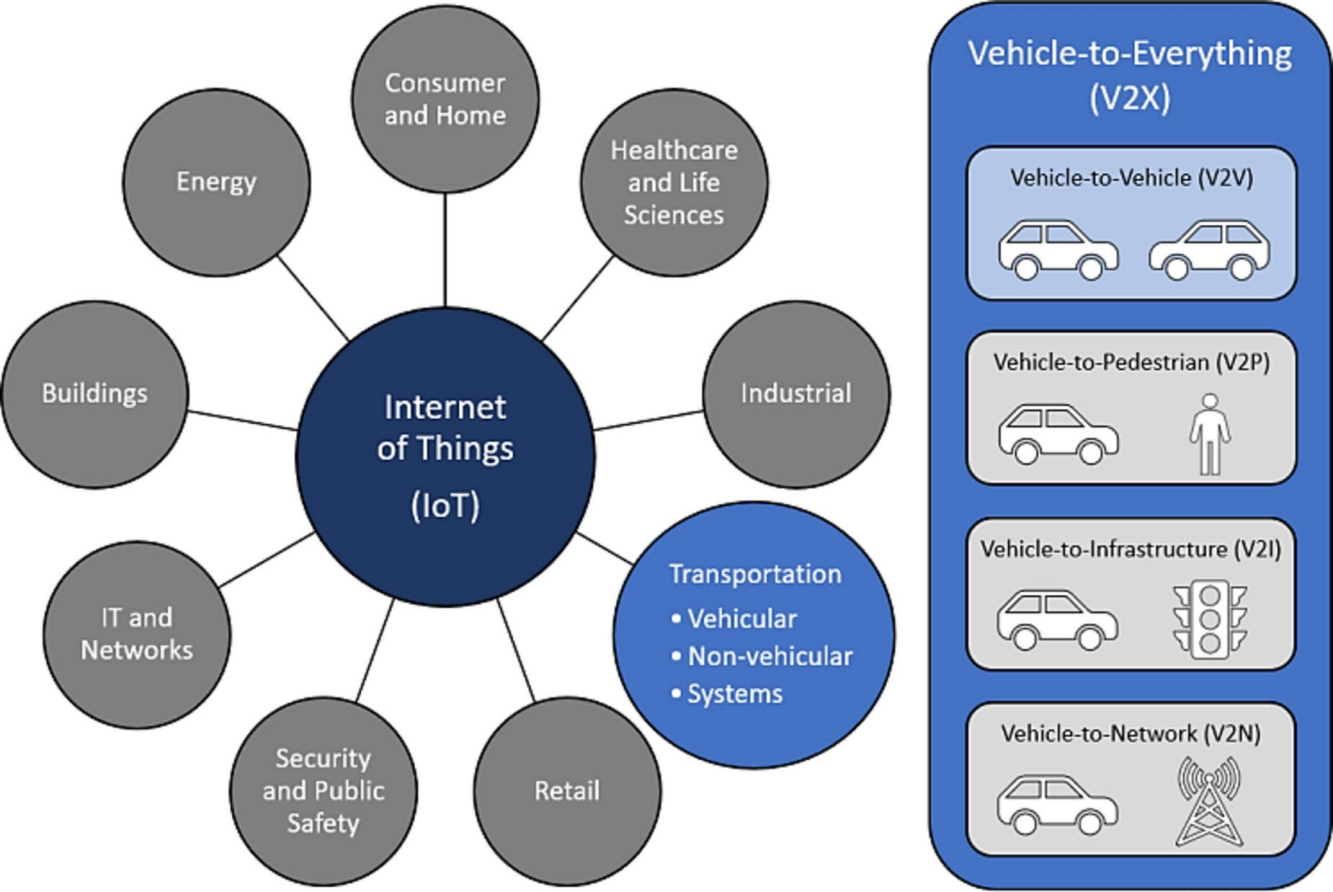
Diagram of the IoT sectors and the association among V2V and V2X.

More and more AVs will be found on the roads of the world as AVs enter the commercial sector and develop towards complete autonomy^1^. Cameras and LiDAR are only two examples of the sensors that modern AVs use to keep an eye on the road and drive safely and effectively^2^. A communication, however, permits AVs to share information with one another in the event that other AVs are already on the road. Safer and more efficient driving is possible because to V2V communication amongst AVs in real time^3^. If a person is in the road but invisible to one autonomous vehicle (AV), V2V communication may alert other AVs to the pedestrian's location so that they could all see the pedestrian. (V2I) communication enables AVs to exchange data with other computing devices, such as roadside equipment. The V2X concept includes both vehicle- connections. As AVs become more commonplace, V2V and V2I connections can enhance standard AV operations. They will also help to link autonomous vehicles (AVs) to the broader (IoT) (Fig. 1)^4^.

The phrase "Internet of Things" (IoT) describes a network of "things" (loaded with software, actuators, and sensors) that are connected over the web in order to collect and exchange data^5,6^. According to the (IDC), the market for Internet of Things devices is expected to reach $8.9 trillion and 41 billion units will be in operation by 2020^6^. Specifically, the Internet of Things (IoT) has been implemented in (STS) to improve traffic management for the general public^7^. There have been several large-scale STS applications made possible by the Internet of Things. For instance, a known as the "internet of vehicles," is created by collecting transportation data from millions of vehicles that provide a wide variety of information sources. With the use of IoT, projects like traffic control rooms systems have contributed to the evolution of the STS recommendation system, which is based on predictive calculation of user actions^8^.

Cyber-physical systems (CPS) are a key component of smart transportation, which is being propelled by the Internet of Things (IoT) and integrated with CPS thanks to open network technologies that create linkages between diverse bulges of transport, instantiated as a scheme of schemes^9^. However, due to its heterogeneity, difficulty, and decentralization^10^, cybersecurity and 5g-enabled network connection pose significant problems for an IoT-driven, CPS-embedded system.

To begin, a wide range and abundance of data are produced while utilizing the IoT hardware in the smart transportation system. If properly utilized, these data have the potential to enhance traffic monitoring and bring the transportation system closer to full intelligence^11^. Traditional security methods are unable to be deployed on IoT systems because to data explosion in the STS and the restricted memory space of nodes, which is insufficient for the data needs of better security processes. Concerns around data leakage and system breaches^12^ are another consequence of data explosion problems. Second, the IoT nodes may independently exchange data with one another through device-to-device interaction. Making good use of data enhances scheme execution by providing relevant domain knowledge^13^. The diversity, complexity, and decentralization of STS allowed by IoT make data exchange between them a difficult problem. Third, due to the real-time transfer of IoT traffic and the quick reaction needs of smart applications, the network connection is of vital significance for the STS. communication costs are the primary issues plaguing today's Internet of Things-enabled smart transportation security system^14^. It's also possible that the wireless network nodes implanted in the STS aren't properly protected from physical harm.

Existing solutions that are readily obtainable for 'traditional' schemes, in this case to resolving difficulties associated with CPS, may not be sufficient to overcome these obstacles. This is due to the fact that most recent developments in CPS and IoT have focused on data and intelligent devices, with a particular emphasis on exchanges of information at the micro level and transfers of data between nodes. Geographical indicators that have far-reaching effects on the rollout of transportation sensor networks are examples of the kinds of macro concerns that must be put up in connection to geospatial and public infrastructural setups. According to the research, artificial intelligence (AI) and geospatial security management work hand in hand to tackle the aforementioned difficulties. The paper employs a hybrid optimization approach to optimize SVM communication. The experimental evaluation compares the geolocation data to different ML models to ensure its efficacy.

This section of the paper is structured as shadows: The relevant literature is obtainable in "Related works" section, and the suggested model is summarized and deliberated in "Proposed methodology" section. "Results and discussion" section contains the validation analysis and its comments. "Conclusions" section delivers a summary and conclusion of the study.

## Related works

Car-following models based on dynamic topology have been proposed by Liu et al.^15^. We then investigate the stability analysis technique. Finally, we show the consequences of a series of arithmetical simulations done to investigate platoon stability for various communications topology situations, using the (DC-IDM) as an instance. The consequences reveal that a decrease in stability occurs when there is a breakdown in communication, but that it is restored when additional information is gathered from cars ahead and the required time headway is increased. In addition, the research investigates the minimum percentage of communication failures that must occur before stability is lost for various values of the driving parameters.

The interaction between (CVs) and autonomous vehicles (AVs) at crosswalks has been studied by Taima and Daimon^16^. Every 0.5 s, we collect the (x and y coordinates), and then use this data to do a hot spot analysis of the pedestrian's movements. The Wizard of Oz technique is used to regulate the behavior of the CV and AV, both of which are Toyota Prius (ZVW30) models. Each CV and AV experiment has 38 individuals and takes place on a public road in Japan. After talking to the CV or AV, the participants go to the other side of the road. The findings demonstrate that pedestrians can cross the street earlier when interacting with the CV compared to the AV. The walkers who interact with the CV opt to cross the street before the CV stops, while the pedestrians who interact with the AVs prefer to cross the street after the AV stops, as shown by the hot spot study. Perceived safety is found to have little effect on pedestrian behavior; as a result, drivers' communiqué and the external human–machine interface should prioritize early perceptions of safety over increasing perceptions of safety.

A unique cyber-physical framework for optimum multi-lane highways has been created by Sakaguchi et al.^17^. To provide a safe and orderly lane shift or merging, cars are organized into groups using a receding horizon control (RHC) strategy. We assume that all vehicle data is stored in a cloud-based computer an optimization issue is performed in order to determine optimal group speeds and locations for each vehicle. The cars' local controllers then decide on the appropriate control acceleration for each vehicle to take in order to maintain the planned trajectory and keep everyone on the road safe. The efficacy of the projected traffic coordination system was assessed in a real-world, multi-lane road environment. The results demonstrate that the suggested framework considerably reduces velocity, and trip time for cars operating in a variety of traffic conditions. The computational requirements of our approach are so low that it may be deployed on the cloud.

Sensor data vehicular network including vehicle- communication is something that Song et al.^18^ have thought about. Bidirectional Feedback Noise Estimation (BiFNoE) is a method we propose in which sensor measurement data from cars is gathered and stored in a cache on an edge server. Each vehicle's distributed sensing is improved with reduced communication costs thanks to the edge's alternating noise and target estimations in double dynamic sliding time frames. We simulate an application scenario to test the suggested algorithm and data distribution approach, and find that with just 12 kbps of uplink and 28 kbps of downlink bandwidth, the enhanced by roughly 80%.

To dampen the tremors of traffic, Shi et al.^19^ present a distributed longitudinal control technique for CAVs based on (DRL) in the event of a communication breakdown. To mimic real-world the DRL training environment incorporates signal-interference-plus- communiqué. In order to mitigate the jarring effects of the dynamic IFTs on the control signal, a dynamic information fusion technique has been developed. To establish the consensus equilibrium in the multi-agent system, each CAV controlled by the DRL-based agent was designed to receive the state information of the downstream CAVs in real time and conduct longitudinal actions. To test the control performance, oscillation capabilities of our proposed algorithm and adjustment mechanism, we conduct simulated experiments.

Xiao et al.^20^ have concentrated on linked automated vehicles across resource-constrained networks. First, we develop a fundamental platoon-based control framework that accounts for a wide variety of conditions, such as sporadic data sampling and transmission, varying spacing regulations, and a number of possible topologies for communication between vehicles and their leaders. To avoid exhausting the network's bandwidth, an adaptive Zeno-free event-triggered communication mechanism is industrialised to synchronise the timing of data sampling and transmission across vehicles in a particular scenario. Then, a scalable co-design approach is used to create the necessary adaptive condition. Since the proposed platooning control solution does not require information about the global communiqué topology or the size of the platoon, it has the potential to be implemented manner. Finally, to ensure the accuracy of the generated method, we conduct a series of numerical case studies. When it comes to controlling CAVs in a platoon when they're all experiencing occasional denial-of-service (DoS) assaults, Ge et al.^21^ have you covered. To begin, a heterogeneous and uncertain model of a vehicle's longitudinal dynamics is described so as to account for variables such as varying vehicle weights and engine inertial postponements, unknown forces, and a moving platoon commander. The goal is to maintain individual vehicle stability, assault all at once, therefore a robust and safe distributed control rule is built. To further ensure the stability, safety, and scalability of the platoon in the face of DDoS attacks, a numerically efficient offline design approach is created to determine the optimal control rule for the platoon. Finally, a large number of numerical tests are presented to prove that the suggested platooning technique works.

It is the job of Madhubabu et al.^22^ to determine the optimal and fastest path from A to B. The BHGWO model that has been suggested handles this. The purpose of this article is to present a VANET route selection algorithm. In order to choose the best paths or routes, it is important to record the vehicle information between the source and the destination. The information about congestion and mobility is then saved in the IoT. Before choosing the best way, all of the potential paths are listed out. To address this issue, we provide the Bat Hybridised Grey Wolf Optimisation (BHGWO) technique, a novel hybrid optimisation model that essentially merges the Bat Optimisation Algorithm (BOA) with the Grey Wolf Optimisation (GWO) strategy. In order to determine the convergence analysis of the recognised BHGWO model, the usual schemes ACO, GA, GWO, CSA, and BOA were used in chronological order. In addition, the performance was evaluated in terms of energy, congestion, delay, and vehicle count, with 50, 100, 150, and 200 vehicles used for each category, respectively.

## Proposed methodology

- Taking into account the aforementioned situations and the research conducted in the arena of vehicles, it is abundantly clear that there is a essential to work on geo how to effectively integrate geo spatial data into the existing infrastructure of the field.
- Geospatial data processing, analysis, and visualization.
- Better mobility is possible through data sharing between humans and autonomous cars.
- The planned study will primarily focus on figuring out how to effectively relay geospatial data to the next generation of transport machines.

### Geo Spatial Data

The term "Geo Spatial Data" refers to information of a static or dynamic type concerning an object's location on Earth's surface.

In this case, information on the cars will be stored and made available to other parties. Since most things in the real world are either always changing or always staying the same, enormous amounts of geo spatial data are always being produced. Autonomous transportation systems need a great deal of geospatial data, most of which has already been collected. The largest obstacle, though, is having the autonomous car really make use of this information.

Autonomous cars can use geospatial data to determine the quickest route, name nearby landmarks, and relay this information to the next available vehicle^23^. The suggested process would begin by collecting all available geospatial data from sources. This data may be fully or partially organized. When we receive the information, we'll save it in the proper format. In order to supply the necessary information for the driverless cars, Google maps will be considered alongside geo spatial data.

All cars' geolocation information will be captured in real time according to the suggested manner. In addition, this information will be shared among autonomous cars via peer-to-peer networking to improve mobility and delay tolerance. Choosing the optimum route to minimize congestion, natural catastrophes, needless delays, etc., is the most significant consideration in vehicular ad hoc networks^24^.

The suggested study focuses mostly on already-existing technology; for instance, suppose an autonomous car is traveling from point A to point B. Most self-driving cars will use Google Maps, where data on latitude and longitude may be found. The same will be able to use autonomous cars to get from point A to point B. Here, the largest challenge is informing all autonomous cars headed in the same direction of any major delays they may encounter along the route, such as heavy traffic or a natural disaster. Better mobility might be possible if more vehicles than just this one were able to interact with one another.

### Communication of vehicles using machine learning

Vapnik's suggested version of Support Vector Machines was used, which employs a loss function that is insensitive to the value of in order to address the regression issues at hand. The following equation describes the general estimating function of SVM for regression:

1 $$f\left(x\right)=\left(w.\Phi (x)\right)+b$$

where $w\subset {R}^{n}$, $b\subset R$ and the $\Phi$ denotes a alteration from ${R}^{\text{n}}$ to space. The impartial is to discovery the standards of *w* and *b* factors such that standards of *x* can be distinct by minimalizing the reversion risk ${R}_{\text{regression}}$.

2 $${R}_{\text{regression}}\left(f\right)=C\sum_{i=0}^{{\ell}}\Gamma \left(f\left({x}_{i}\right)-{y}_{i}\right)+\frac{1}{2}{\Vert w\Vert }^{2}$$

where Γ(.) signifies the cost purpose, C signifies a continuous which describes consequences to the projected errors and w is the vector:

3 $$w=\sum \limits_{i=1}^{{\ell}}\left({a}_{i}-{a}_{i}^{*}\right)\Phi ({x}_{i})$$

where ${a}_{\text{i}}$ and ${a}_{i}^{*}$ are multipliers, if you will. By applying Eq. (3) to the solution of Eq. (1), we get the following form of Eq. (1):

4 $$f\left(x\right)=\sum_{i=1}^{{\ell}}\left({a}_{i}-{a}_{i}^{*}\right)\left(\Phi \left({x}_{i}\right).\Phi (x)\right)+b$$

And lastly, by replacement the the kernel function, $K({x}_{i},{x}_{j})$ we have:

5 $$f\left(x\right)=\sum_{i=1}^{{\ell}}\left({a}_{i}-{a}_{i}^{*}\right)k\left({x}_{i},{x}_{j}\right)+b$$

The ε- The following equation expresses the insensitive cost function:

6 $$\Gamma \left(f\left(x\right)-y\right)=\left\{\begin{array}{ll}\left|f\left(x\right)-y\right|-\varepsilon, & \quad for \left|f\left(x\right)-y\right|\ge \varepsilon \\ 0 & \quad otherwise\end{array}\right.$$

Linear, Gaussian, polynomial, and the Radial (RBF) kernels are the four most common kinds of kernel functions employed by Support Vector Machines. The dot product of two vectors is the operation specified by the linear kernel.

7 $$K\left({x}_{i},{x}_{j}\right)=\langle {x}_{i},{x}_{j}\rangle$$

The Gaussian Kernel function is distinct by the subsequent equation:

8 $$K\left({x}_{i},{x}_{j}\right)={e}^{-\frac{1}{{2\sigma }^{2}}{\Vert {x}_{i}-{x}_{j}\Vert }^{2}},\sigma >0$$

where sigma (σ) is a limit be contingent from gamma (γ) limit is distinct as:

9 $$K\left({x}_{i},{x}_{j}\right)={\left(\langle {x}_{i},{x}_{j}\rangle +r\right)}^{d},d>0,r\ge 0$$

where *d* signifies the polynomial grade, and *r* is a fixed limit.

The RBF Kernel is distinct by the subsequent equation:

10 $$K\left({x}_{i},{x}_{j}\right)={e}^{-\gamma {\Vert {x}_{i}-{x}_{j}\Vert }^{2}},\gamma >0$$

### The proposed new w-SVM kernel

The projected kernel, self-possessed of two factors, the ${W}_{\text{m}}$ factor and the *KBEST* $(x, x^{\prime})$ which couriers the optimal kernel originate by applying hybrid optimization. The ${W}_{\text{m}}$ factor couriers the weight matrix shaped by hybrid optimization’s* weight vectors ${W}_{\text{i},\text{j}}$ and ${W}_{\text{i},\text{j}}^{T}$. The motive for increasing the weight vectors ${W}_{\text{i},\text{j}}$ and ${W}_{\text{i},\text{j}}^{T}$ is to construct a symmetrical SVM kernel that contents the circumstances of Mercer’s theorem.

11 $${K}_{w-SVM}\left(x,{x}^{\prime}\right)=\left({W}_{i,j} \cdot {W}_{\text{i},\text{j}}^{T}\right){K}_{BEST}(x,x^{\prime})$$

12 $${K}_{w-SVM}\left(x,{x}^{\prime}\right)={W}_{m}{K}_{BEST}(x,x^{\prime})$$

- ${W}_{i,j}$ is the matrix of the hybrid optimization.
- ${W}_{\text{i},\text{j}}^{T}$ is the move last weight of the hybrid optimization.
- ${K}_{BEST}(x,x^{\prime})$ is the finest kernel found by smearing optimization.
- *x*, *x*′ are the input vectors.
- ${W}^{\text{m}}$ signifies the matrix ${W}_{i,j}$ ⋅ ${W}_{\text{i},\text{j}}^{T}$

As a positive semi-define, the hybrid kernel described here fulfills Mercer's theorem. Since the RBF kernel performed best in this case study, we will use it to represent the SVM.

13 $${K}_{BEST}\left(x,{x}^{\prime}\right)={K}_{RBF}(x,x^{\prime})$$

The suggested W-SVM kernel in this application is stated by combining Eqs. (12) and (13), as shown below:

14 $${K}_{w-SVM}\left(x,{x}^{\prime}\right)={W}_{m}{K}_{RBF}(x,x^{\prime})$$

where ${K}_{RBF}(x,x^{\prime})$ is the RBF kernel function. The primary goal is to import the optimum model's weight vector into the suggested W-SVM kernel, which will subsequently be utilized to make predictions. The first step involved building support vector machine models through a combination of hyperparameter hybrid optimization and validation for each traditional SVM kernel function. Next, the suggested model-based optimized kernel function's converted weight vector was incorporated into the proposed W-SVM kernel's development.

### Hyper-parameter tuning using hybrid model

#### Grey wolf optimizer (GWO)

The GWO algorithm, initially projected by Mirjalili^23^, is based on the idea that a pack of grey wolves, consisting of anything from five to twelve members, has an established hierarchy and hunting style. To represent the many tiers of authority within a pack of wolves, each pack is subdivided into alpha, beta, delta, and omega packs. Wolves defer to their alpha leaders for important choices including when to rise, where to hunt, and where to sleep. The beta wolf's major role is to offer reaction recommendations to the alpha wolf while the latter is making a choice. The omega wolves are under the command of the caretakers, hunters, elders, sentinels, and scouts of the delta wolf pack. The grey wolf with the lowest social status is an omega, and it must constantly take second place to higher-status wolves. Wolf packs have leaders known as alpha, beta, and delta; omega wolves are expected to follow their lead during hunts. Wolves go through three primary phases during a hunt: tracking and approaching their prey, besieging and harassing it until it stops moving, and then attacking. The GWO algorithm was developed with the wolves' hunting strategy and social structure in mind. Here we discuss the formalization of the GWO algorithm in mathematics.

To quantitatively explain the encircling behavior of grey wolves, Mirjalili proposes two equations^23^.

15 $$\left|\left(t\right)-\overrightarrow{X}\left(t\right){\overrightarrow{X}}_{P}.\overrightarrow{C}\right|=\overrightarrow{D}$$

16 $$\overrightarrow{D}\left(t\right)-\overrightarrow{A}.{\overrightarrow{X}}_{P}\left(t+1\right)=\overrightarrow{X}$$

where $\overrightarrow{A}$ and $\overrightarrow{C}$ are constant vectors while t demonstrations the current iteration. $\overrightarrow{X}$ ***P*** vector is related to the site of the prey and $\overrightarrow{X}$ Site of grey wolf is shown as a vector. Following Eqs. (17) and (18), we may determine the values of the vectors A and C.

17 $$\overrightarrow{A}=2\overrightarrow{a}.{\overrightarrow{r}}_{1}-\overrightarrow{a}$$

18 $$\overrightarrow{C}=2.{\overrightarrow{r}}_{2}$$

where elements of $\overrightarrow{a}$ are linearly reduced from 2 to 0 over the course of repetitions and ${\overrightarrow{r}}_{1}$, ${\overrightarrow{r}}_{2}$ are random vectors in [0, 1].

The grey wolf has the ability to locate its victim, then encircle and kill it. The alpha wolf normally directs the hunt, although the beta and delta wolves will sometimes help out. Since this is the case, we will initially focus as the top three possible solutions for mathematically reproducing grey wolf hunting behavior. This is because omega wolves are more likely to recognize the general area where their prey is hiding. In the current study, the top three answers were recorded and modeled mathematically, while the remaining agents were compelled to adjust their locations accordingly. The following equations are proposed for this purpose:

19 $$\left|-\overrightarrow{X} {\overrightarrow{X}}_{\delta }.{\overrightarrow{C}}_{3}\right|={\overrightarrow{D}}_{\delta },\left|-\overrightarrow{X} {\overrightarrow{X}}_{B}.{\overrightarrow{C}}_{2}\right|={\overrightarrow{D}}_{\mathcal{B}},\left|-\overrightarrow{X} {\overrightarrow{X}}_{\propto }.{\overrightarrow{C}}_{1}\right|={\overrightarrow{D}}_{\propto }$$

20 $$-{\overrightarrow{A}}_{3}.{\overrightarrow{D}}_{\delta }{\overrightarrow{X}}_{\delta }={\overrightarrow{X}}_{3},-{\overrightarrow{A}}_{2}.{\overrightarrow{D}}_{B}{\overrightarrow{X}}_{B}={\overrightarrow{X}}_{2}-{\overrightarrow{A}}_{1}.{\overrightarrow{D}}_{\propto }{\overrightarrow{X}}_{\propto }={\overrightarrow{X}}_{1}$$

21 $$\overrightarrow{X}\left(t+1\right)=\frac{{\overrightarrow{X}}_{1}+{\overrightarrow{X}}_{1}+{\overrightarrow{X}}_{1}}{3}$$

where the best solution is supposed as keys are called beta (β) and delta (δ), correspondingly. The rest of the other solutions are documented to be omega (ω).

The value of a can be decreased to represent getting closer to the prey in a mathematical model. For random values of A in the interval [− 1,1], the explore agent's next possible location is anywhere between its current location and the location of the prey.

When searching for food, wolves of dissimilar packs divide up into smaller groups known as alpha, beta, and delta packs. By letting A take on random values larger than 1 or smaller than -1, we can represent the separation mathematically and cause the explore go-betweens to diverge from the prey. Grey wolves, according to their value system, must also withdraw from their prey in order to discover healthier alternatives. |A|> 1.

$\overrightarrow{C}$ vector is additional constituent of the GWO procedure that covers chance values in [0,2] and consequently delivers accidental weight for prey. As a consequence, the consequence of prey in categorizing the coldness is stochastically highlighted or value of $\overrightarrow{C}$ vector is correspondingly greater than 1 and less than − 1.

#### Elephant herding optimization

Wang et al.^23^ initially presented the elephant herding optimization (EHO) method. Wild elephants are known to congregate often. Female elephants tend to remain within their families, which are led by the eldest matriarch. Males, on the other hand, prefer to leave the tribe once they reach adulthood and have the amazing capacity to use herding behavior of elephants to communicate with their tribe members via low-frequency vibrations, a global optimization issue answered by ideal laws that we discuss further below.

- Elephants are social animals, and they tend to congregate in groups with a predetermined number of members.
- A percentage of young male elephants in each generation decide to strike off on their own.
- A matriarchy, often the oldest and most experienced elephant in the tribe, takes charge and makes all important decisions on behalf of the clan.

##### Clan updating operator

The next stop for every elephant in clan c_i is decided by matriarch ${c}_{i}$, as is the case with all tribes. It can be changed so that the elephant j in clan ${c}_{i}$,becomes:

22 $${X}_{new,{c}_{i,j}}={X}_{{c}_{i,j}}+\alpha \times \left({X}_{best,{c}_{i}}-{X}_{{c}_{i,j}}\right)\times r$$

where ${X}_{new,{c}_{i,j}}$ and ${X}_{{c}_{i,j}}$ are lately studied and old site clan ${c}_{i}$, correspondingly. α ∈ [0,1] is a scale factor that agrees the effect of materfamilias ci on ${X}_{{c}_{i,j}}$. ${X}_{best,{c}_{i}}$ proves matriarch ${c}_{i}$, which is the best elephant distinct in clan ${c}_{i}$. *r* ∈ [0, 1]. distribution is utilized in this study.

23 $${X}_{new,{c}_{i,j}}=\beta \times {x}_{center,{c}_{i}}$$

where β ∈ [0, 1] is a issue that controls the result of the ${X}_{new,{c}_{i,j}}$ on ${X}_{new,{c}_{i,j}}$. It can be seen that, the new separate ${X}_{new,{c}_{i,j}}$ in Eq. (23) is shaped by the data calm by entirely the elephant individuals in clan *ci*. ${x}_{center,{c}_{i}}$ is be assessed as:

24 $${x}_{center,{c}_{i,d},}=\frac{1}{{n}_{ci}}\times \sum_{j=1}^{{n}_{ci}}{X}_{ci,j,d}$$

where 1 ≤ *d* ≤ *D* postulates the *d*th dimension, and *D* is its entire dimension. *nci* shows the sum of elephant separate *xci*,. The center of clan *c*, ${x}_{center,{c}_{i,d},}$ can be assessed through *D* evaluations rendering to Eq. (24).

##### Separating operator

Adult male elephants in elephant groups often go out and start new families on their own. To further develop the research for the EHO technique, the male elephants always use the separator worker in each cohort, as shown in Eq. (25). This parting procedure may be modelled on the separator operator while tackling optimization issues.:

25 $${X}_{worst,ci}={X}_{min}+\left({X}_{max}-{X}_{min}+1\right)\times rand$$

where ${X}_{min}$ and ${X}_{max}$ are lower site of elephant separate, respectively. ${X}_{worst,ci}$ is the worst elephant separate in clan *ci*. *rand* ∈ [0, 1] is a chance quantity uniformly dispersed in the variety [0, 1]. Therefore, algorithm 3 can perfect the unravelling operative.

#### Proposed GWOEHO

In this section, a novel hybrid method is presented by combining aspects of the GWO algorithm with those of the EHO algorithm. As mentioned before, the GWO algorithm takes cues from the hunting strategies of wolves. Despite this, the isolation of male elephants from the group and the clan structure in which they function have served as inspiration for the development of optimization methods. The hybrid GWOEHO algorithm utilizes elephant clan dynamics to split the wolf population into manageable packs. After identifying the wolves in each pack, the GWO algorithm proposes a procedure for changing the status of the wolves based on this information. Alpha, beta, and gamma wolves around the pack have all had their status updated in the text.

Then, a new separation operator is introduced to characterize the partitioning procedure. In the new operator eht dna proposal, we pick one or two wolf packs at random and then use a formula to eliminate from these packs.

26 $${X}_{worst,ci}=t\times \left({x}_{max}\right)\times {(-1)}^{Cindex}$$

The variable t in the overhead equation is abridged using the Eq. (27) and relative to Maxiter from the worth of one to zero.

27 $$t=1-iter\times \left(\frac{2}{{Max}_{iter}}\right)$$

$$Iter\in [0,{Max}_{iter}/2]$$

**Fig. 2:**
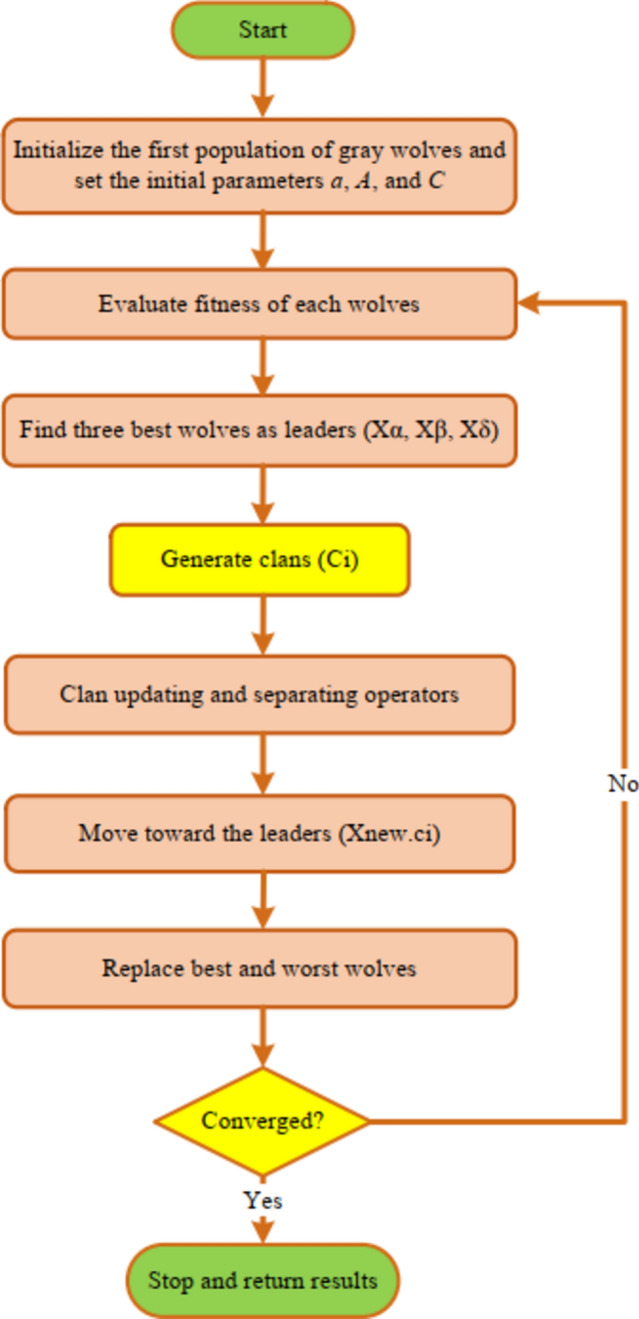
Flowchart of projected GWO-EHO algorithm.

In the overhead relation, the present security iter ${Max}_{iter}$ is equal to the extreme repetition cycle of the procedure and *xmax* of Cindex = 1 or cindex = 2. Flowchart of the model is mentioned in Fig. 2.

## Results and discussion

### Performances metrics

Apiece algorithm is assessed in terms of accuracy, F1-measure. An ideal machine learning algorithm should achieve with low false alarm rate.

Accuracy is totaled as shadows:

28 $$Accuracy=\frac{TP+TN}{TP+TN+FP+FN}$$

As shown above, TP represents a correctly identified malicious flow, TN indicates a normally identified flow, FN represents a wrongly identified flow of normal traffic, and FP represents a wrongly identified attack flow.

How to Determine the Rate of False Alarms.

29 $$False Alarm Rate=\frac{FP}{TN+FP}$$

Precision is computed as following

30 $$Precision=\frac{TP}{TP+FP}$$

Recall is calculated as follows

31 $$Recall\frac{TP}{TP+FN}$$

Lastly, the F1-measure is subtracted as shadows:

32 $$\text{F}1{\text{-}}\text{measure}=2\times \frac{Precision\times Recall}{Precision+Recall}$$

The answers of the evaluation are portrayed in below section.

### Analysis of proposed machine learning

Tables 1 and 2 presents the comparative analysis of various ML models by using two different kinds of ratio on data.

**Table 1:** Analysis of proposed model for 60–40%.

ML techniques	Accuracy (%)	Precision (%)	Recall (%)	F1 score (%)
MLP	88.03	79	72	72
ELM	90.32	82	73	72
SVM	91.64	83	76	75
Proposed	94.76	86	80	80.06

**Table 2:** Analysis of proposed model for 80–20%.

ML techniques	Accuracy (%)	Precision (%)	Recall (%)	F1 score (%)
MLP	93.1	92.00	93.00	93.00
ELM	94.4	78.33	81.86	80.05
SVM	95.01	95.46	94.51	94.98
Proposed	96.56	96.76	95.54	95.67

**Fig. 3:**
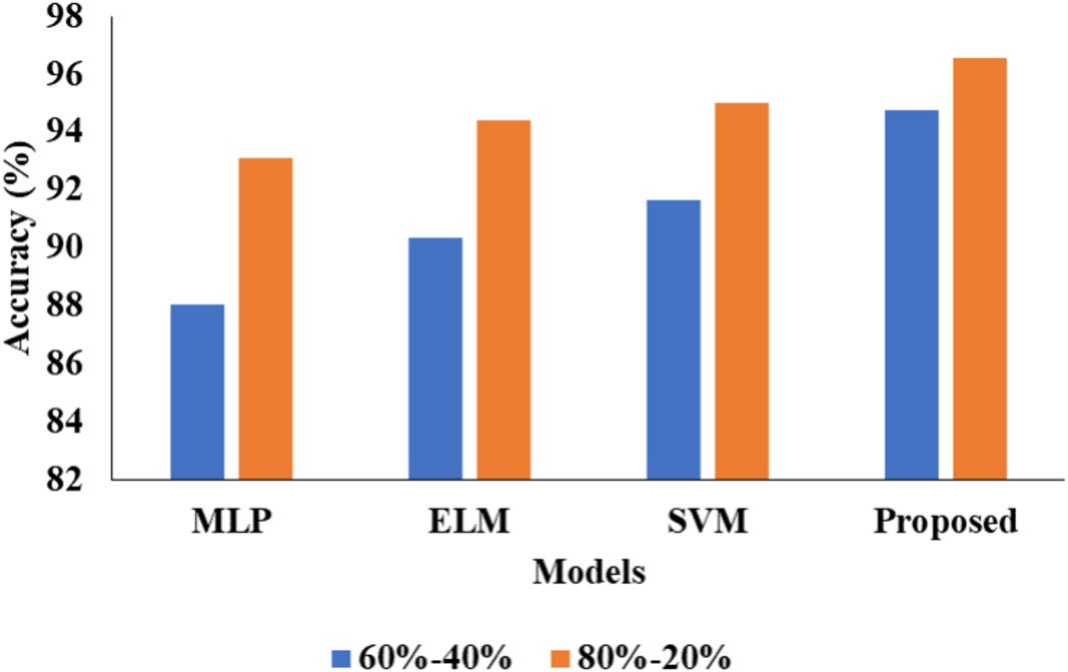
Accuracy analysis.

**Fig. 4:**
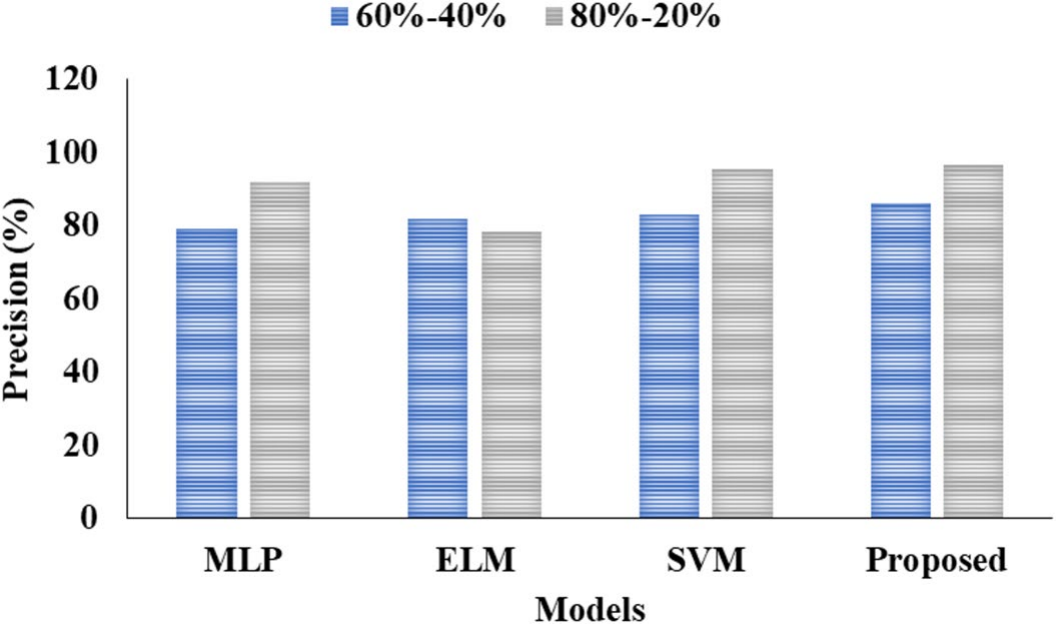
Precision comparison.

**Fig. 5:**
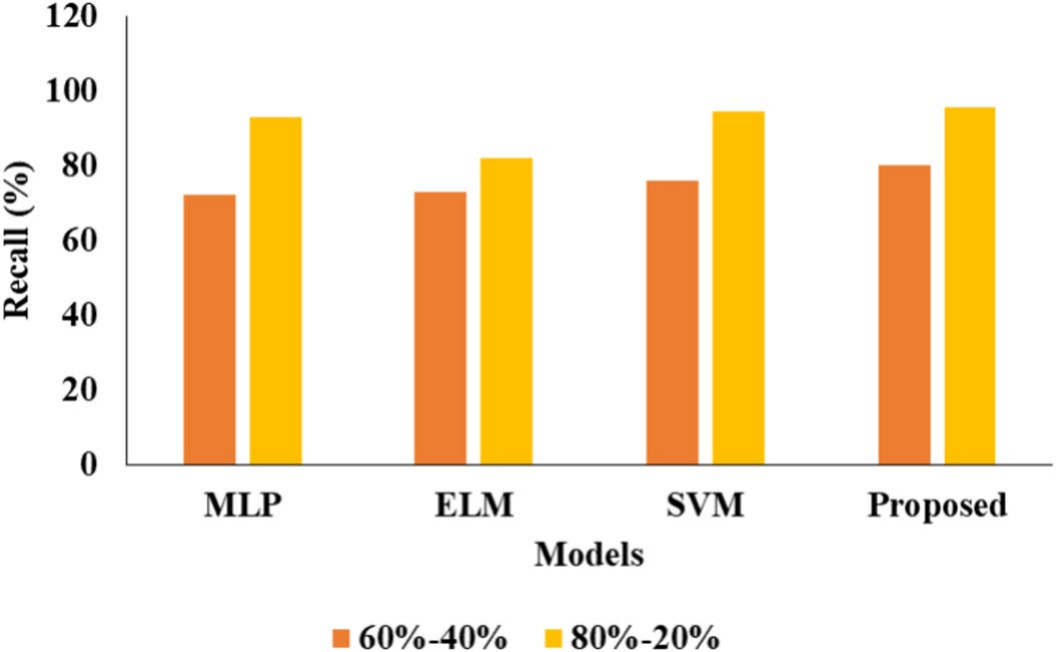
Recall Presentation.

**Fig. 6:**
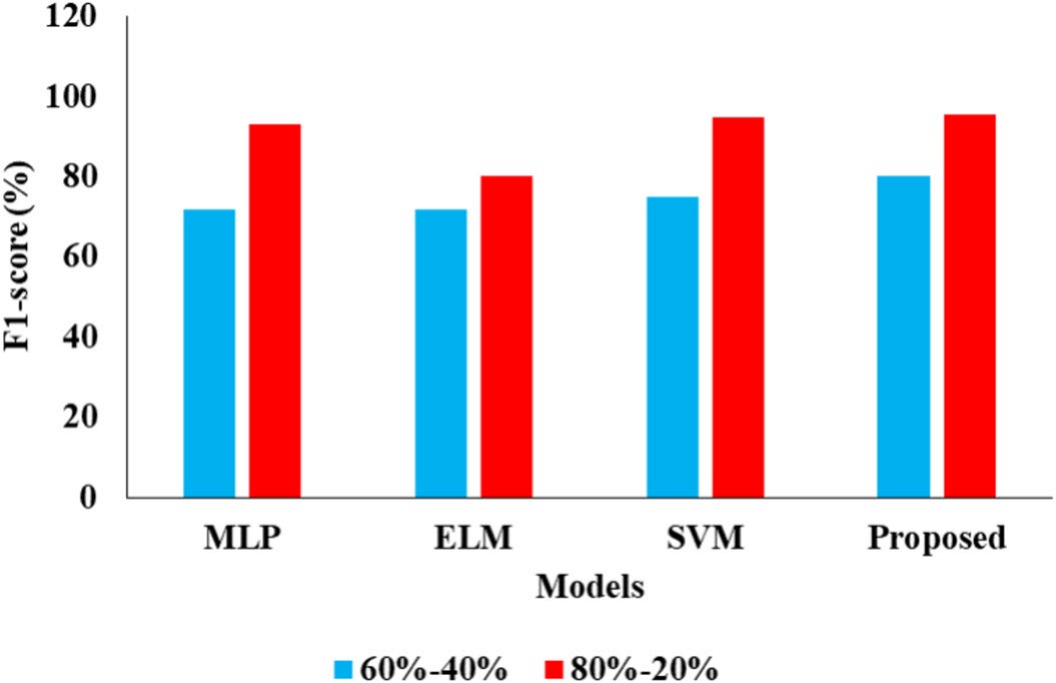
Graphical Comparison for F1-score.

Table 1 above. Several techniques, including MLP, ELM, and SVM using the proposed methodology, were employed in this study. The accuracy, precision rate, recall, and F1-score were all obtained using the MLP approach, with results of 88.03, 79, 72, and 72, respectively. The accuracy, precision rate, recall, and F1-score were all obtained using the ELM approach, with results of 90.32, 82, 73, and 72, respectively. The accuracy of the SVM approach was determined to be 91.64 as shown in Fig. 3, in addition to the precision rate of 83 shown in Fig. 4, recall of 76, and F1-score of 75 for everyone. The accuracy and precision of the suggested methodology's approach were 94.76 and the precision rate as 86 and the recall as 80 shown in Fig. 5 and in conclusion the F1-score as 80.06 individually shown in Fig. 6.

Table 2 above illustrates the investigation of the projected model for the 80–20%. Various approaches, including MLP, ELM, and SVM with proposed methodology, were employed in this analysis. The MLP approach yielded results with an accuracy of 93.1, a precision cost of 92.00, a recall value of 93.00, and an F1-score value of 93.00 in that order. An alternative approach to ELM yielded results of 94.4 percent accuracy, 78.33 percent precision, 81.86 percent recall, and 80.05 percent F1-score, in that order. Another SVM approach produced results including accuracy of 95.01, precision rate of 95.46, recall value of 94.51, and F1-score value of 94.98 in that order. The proposed method's final results showed accuracy of 96.56, precision charge of 96.76, recall value of 95.54, and F1-score charge of 95.67, respectively.

### Analysis of models for communication network

**Fig. 7:**
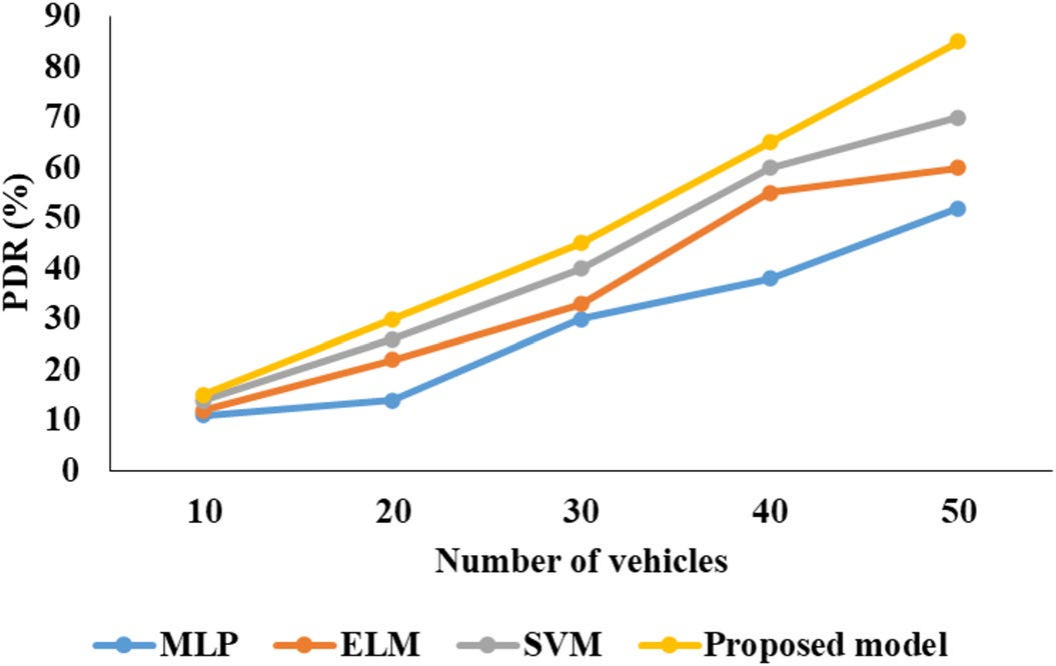
PDR analysis.

**Fig. 8:**
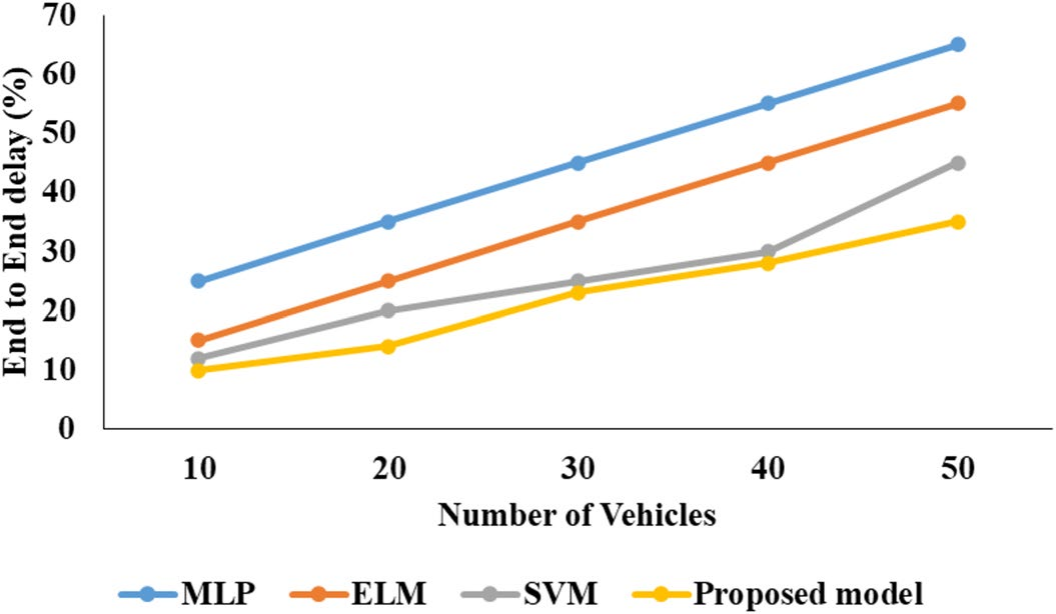
End to end delay.

**Fig. 9:**
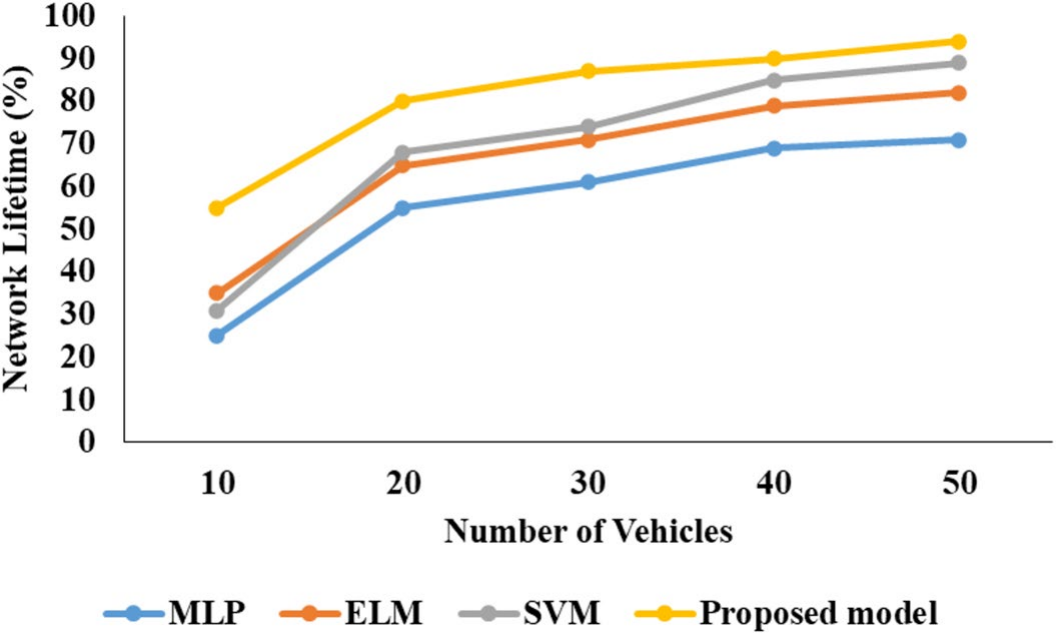
NLT comparison.

Figures 7, 8, 9 presents the graphical comparison for proposed model for V2V communication analysis.

In the above Fig. 7 represent that the PDR investigation of projected perfect with other techniques as MLP, SVM and projected model. In this investigation the proposed model reached better PDR presentation than associated methods.

In the above Fig. 8 represent that the End-to-end delay investigation of proposed model with other techniques as MLP, SVM and projected model. In this investigation the proposed model reached better End to end delay performance than compared methods.

In the above Fig. 8 characterize that the NLT Comparison analysis of projected model with other techniques as MLP, SVM and proposed model. In this investigation the proposed model reached better NLT presentation than associated methods.

## Conclusions

In the last several decades, advances in machine learning have facilitated the widespread use of intelligence across a variation of spheres of human activity. Improved outcomes in the communication process for autonomous cars were found using a hybrid SVM kernel, which was built from scratch using newly created weighted support vector machines. The system will be designed in such a manner that the autonomous cars can interact with each other, allowing for safer and more efficient travel. Using the suggested concept, the cars will be able to exchange information with one another. Communication between cars relies heavily on the ML algorithm. The increased computing power required to run the hybrid algorithm for finding the topology is a kerb of this study. This is because the optimal neural network network topology requires multiple optimizations to be run before the final W-SVM kernel can be constructed, as well as neurons. Other 'depended' SVM kernels will be investigated in future work using alternative classification, which leverage several machine learning methods to get superior prediction outcomes.

## Data Availability

The sample data set information is included in the article that support the findings of this research.

## List of symbols

$\Phi$: Denotes a alteration from to space
${R}^{\text{n}}$: From to space
Γ(.): Signifies the cost purpose
${a}_{\text{i}}$ and ${a}_{i}^{*}$: Are multipliers
$$K({x}_{i},{x}_{j})$$: Kernel function
(γ): Contingent from gamma
(σ): Sigma
*d*: Signifies the polynomial grade
${W}_{i,j}$: Is the matrix of the hybrid optimization
*x*, *x*′: Are the input vectors
${K}_{RBF}(x,x^{\prime})$: Is the RBF kernel function
$\overrightarrow{C}$: Are constant vectors
${\overrightarrow{r}}_{1}$, ${\overrightarrow{r}}_{2}$: Are random vectors
(β): Beta
(δ): Delta
${X}_{min}$ and ${X}_{max}$: Are lower site of elephant separate

## Abbreviations

AV: Autonomous vehicle
EHO: Elephant herding optimisation
GWO: Grey wolf optimizer
IoT: Internet of Things
AI: Artificial intelligence
RHC: Receding horizon control
BiFNoE: Bidirectional feedback noise estimation
BHGWO: Hybridised grey wolf optimisation
SVM: Support vector machine
MLP: Multi-layer perceptron
V2V: Vehicle-to-vehicle
V2X: Vehicle-to-everything
ML: Machine learning
DSRC: Dedicated short-range communication
CAN: Controller area network
